# Supplementary material for: Discovery and application of insertion-deletion (INDEL) polymorphisms for QTL mapping of early life-history traits in Atlantic salmon
Source: BMC Genomics. 2010 Mar 8;11:156. doi: 10.1186/1471-2164-11-156 (PMC2838853; doi:10.1186/1471-2164-11-156)
Supplement: Additional file 2 — Information on developed 76 locus single-run INDEL panel in Atlantic salmon. Information on fluorescence labeling, primer concentrations, PCR pooling and links to alignments, INDEL motifs and GENESCAN (Burge and Karlin 1997) predictions of genes/exons are available in html format. [file 1471-2164-11-156-S2.ZIP › Additionalfile2/table21965.html]

Contig Alignment

```
Base	A B C D   
1	C C . .    
2	A A . .    
3	A A . .    
4	G G . .    
5	A A . .    
6	T T . .    
7	G G . .    
8	A A . .    
9	A A . .    
10	C C . .    
11	A A . .    
12	G G . .    
13	A A . .    
14	A A . .    
15	T T . .    
16	T T . .    
17	C C . .    
18	T T . .    
19	T T . .    
20	C C . .    
21	T T . .    
22	C C . .    
23	A A . .    
24	G G . .    
25	C C . .    
26	A A . .    
27	T T . .    
28	G G . .    
29	T T . .    
30	T T . .    
31	C C . .    
32	G G . .    
33	C C . .    
34	A A . .    
35	G G . .    
36	T T . .    
37	T T . .    
38	G G . .    
39	G G . .    
40	C C . .    
41	C C . .    
42	T T . .    
43	G G . .    
44	T T . .    
45	G G . .    
46	T T . .    
47	T T . .    
48	T T . .    
49	T T . .    
50	G G . .    
51	C C . .    
52	A A . .    
53	G G . .    
54	T T . .    
55	T T . .    
56	G G . .    
57	G G . .    
58	C C . .    
59	C C . .    
60	A A . .    
61	G G . .    
62	G G . .    
63	C C . .    
64	T T . .    
65	C C . .    
66	T T . .    
67	G G . .    
68	A A . .    
69	A A . .    
70	G G . .    
71	T T . .    
72	G G . .    
73	C C . .    
74	T T . .    
75	A A . .    
76	C C . .    
77	A A . .    
78	A A . .    
79	A A . .    
80	T T . .    
81	G G . .    
82	T T . .    
83	G G . .    
84	A A . .    
85	G G . .    
86	T T . .    
87	T T . .    
88	G G . .    
89	G G . .    
90	G G . .    
91	C C . .    
92	T T . .    
93	T T . .    
94	A A . .    
95	T T . .    
96	G G . .    
97	G G . .    
98	G G . .    
99	A A . .    
100	C C . .    
101	C C . .    
102	T T . .    
103	G G . .    
104	T T . .    
105	G G . .    
106	C C . .    
107	T T . .    
108	A A . .    
109	C C . .    
110	A A . .    
111	C C . .    
112	C C . .    
113	A A . .    
114	C C . .    
115	C C . .    
116	G G . .    
117	A A . .    
118	A A . .    
119	G G . .    
120	T T . .    
121	C C . .    
122	A A . .    
123	A A . .    
124	G G . .    
125	T T . .    
126	G G . .    
127	C C . .    
128	A A . .    
129	A A . .    
130	T T . .    
131	G G . .    
132	A A . .    
133	C C . .    
134	G G . .    
135	G G . .    
136	G G . .    
137	G G . .    
138	A A . .    
139	G G . .    
140	A A . .    
141	A A . .    
142	T T . .    
143	T T . .    
144	G G . .    
145	T T . .    
146	T T . .    
147	T T . .    
148	C C . .    
149	A A . .    
150	G G . .    
151	T T . .    
152	G G . .    
153	G G . .    
154	C C . .    
155	G G . .    
156	T T . .    
157	C C . .    
158	G G . .    
159	G G . .    
160	A A . .    
161	G G . .    
162	A A . .    
163	G G . .    
164	G G . .    
165	C C . .    
166	A A . .    
167	G G . .    
168	T T . .    
169	T T . .    
170	A A . .    
171	A A . .    
172	G G . .    
173	G G . .    
174	T T . .    
175	T T . .    
176	G G . .    
177	T T . .    
178	G G . .    
179	G G . .    
180	A A . .    
181	C C . .    
182	A A . .    
183	T T . .    
184	C C . .    
185	A A . .    
186	A A . .    
187	G G . .    
188	A A . .    
189	T T . .    
190	G G G .    
191	A A A .    
192	A A A .    
193	A A A .    
194	G G G .    
195	G G G .    
196	C C C .    
197	T T T .    
198	G G G .    
199	C C C .    
200	C C C .    
201	T T T .    
202	G G G .    
203	G G G .    
204	C C C .    
205	A A A .    
206	G G G .    
207	T T T .    
208	G G G .    
209	A A A .    
210	A A A .    
211	T T T .    
212	G G G .    
213	A A A .    
214	A A A .    
215	T T T .    
216	G G G .    
217	C C C .    
218	A A A .    
219	A A A .    
220	C C C .    
221	A A A .    
222	A A A .    
223	G G G .    
224	A A A .    
225	C C C .    
226	G G G .    
227	A A A .    
228	C C C .    
229	C C C .    
230	A A A .    
231	C C C .    
232	A A A .    
233	A A A .    
234	C C C .    
235	C C C .    
236	G G G .    
237	A A A .    
238	T T T .    
239	T T T .    
240	T T T .    
241	C C C .    
242	C C C .    
243	C C C .    
244	T T T .    
245	T T T .    
246	C C C .    
247	C C C .    
248	A A A .    
249	G G G .    
250	C C C .    
251	T T T .    
252	C C C .    
253	C C C .    
254	A A A .    
255	A A A .    
256	C C C .    
257	C C C .    
258	A A A .    
259	C C C .    
260	A A A .    
261	C C C .    
262	A A A .    
263	G G G .    
264	T T T .    
265	G G G .    
266	T T T .    
267	A A A .    
268	C C C .    
269	A A A .    
270	A A A .    
271	C C C .    
272	A A A .    
273	T T T .    
274	G G G .    
275	A A A .    
276	T T T .    
277	G G G .    
278	A A A .    
279	A A A .    
280	G G G .    
281	G G G .    
282	C C C .    
283	C C C .    
284	T T T .    
285	G G G .    
286	C C C .    
287	T T T .    
288	G G G .    
289	T T T .    
290	G G G .    
291	A A A .    
292	C C C .    
293	A A A .    
294	C C C .    
295	G G G .    
296	G G G .    
297	A A A .    
298	C C C .    
299	C C C .    
300	T T T .    
301	G G G .    
302	T T T .    
303	G G G .    
304	T T T .    
305	A A A .    
306	A A A .    
307	C C C .    
308	G G G .    
309	C C C .    
310	T T T .    
311	G G G .    
312	C C C .    
313	C C C .    
314	C C C .    
315	C C C .    
316	G G G .    
317	G G G .    
318	G G G .    
319	C C C .    
320	C C C .    
321	T T T .    
322	G G G .    
323	C C C .    
324	C C C .    
325	C C C .    
326	C C C .    
327	A A A .    
328	A A A .    
329	A A A .    
330	T T T .    
331	G G G .    
332	A A A .    
333	G G G .    
334	C C C .    
335	A A A .    
336	T T T .    
337	C C C .    
338	C C C .    
339	T T T .    
340	G G G .    
341	C C C .    
342	C C C .    
343	C C C .    
344	C C C .    
345	T T T .    
346	G G G .    
347	G G G .    
348	C C C .    
349	C C C .    
350	C C C .    
351	T T T .    
352	C C C .    
353	G G G .    
354	C C C .    
355	C C C .    
356	A A A .    
357	C C C .    
358	C C C .    
359	C C C .    
360	T T T .    
361	G G G .    
362	A A A .    
363	C C C .    
364	C C C .    
365	A A A .    
366	C C C .    
367	T T T .    
368	G G G .    
369	C C C .    
370	C C C .    
371	T T T .    
372	T T T .    
373	C C C .    
374	A A A .    
375	T T T .    
376	G G G .    
377	A A A .    
378	C C C .    
379	C C C .    
380	A A A .    
381	A A A .    
382	A A A .    
383	G G G .    
384	T T T .    
385	C C C .    
386	C C C .    
387	T T T .    
388	G G G .    
389	G G G .    
390	T T T .    
391	G G G .    
392	T T T .    
393	A A A .    
394	A A A .    
395	A A A .    
396	G G G .    
397	G G G .    
398	G G G .    
399	G G G .    
400	T T T .    
401	G G G .    
402	A A A .    
403	A A A .    
404	G G G .    
405	C C C .    
406	C C C .    
407	C C C .    
408	T T T .    
409	C C C .    
410	T T T .    
411	G G G .    
412	T T T .    
413	G G G .    
414	T T T .    
415	A A A .    
416	G G G .    
417	C C C .    
418	C C C .    
419	T T T .    
420	A A A .    
421	C C C .    
422	A A A .    
423	C C C .    
424	A A A .    
425	C C C .    
426	A A A .    
427	T T T .    
428	G G G .    
429	C C C .    
430	A A A .    
431	T T T .    
432	A A A .    
433	T T T .    
434	G G G .    
435	C C C .    
436	A A A .    
437	G G G .    
438	A A A .    
439	G G G .    
440	G G G .    
441	T T T .    
442	T T T .    
443	G G G .    
444	A A A .    
445	T T T .    
446	A A A .    
447	T T T .    
448	A A A .    
449	C C C .    
450	A A A .    
451	T T T .    
452	G G G .    
453	T T T .    
454	A A A .    
455	G G G .    
456	G G G .    
457	T T T .    
458	T T T .    
459	G G G .    
460	A A A .    
461	T T T .    
462	G G G .    
463	G G G .    
464	T T T .    
465	T T T .    
466	C C C .    
467	A A A .    
468	C C C .    
469	A A A .    
470	C C C .    
471	A A A .    
472	C C C .    
473	C C C .    
474	C C C .    
475	A A A .    
476	C C C .    
477	A A A .    
478	C C C .    
479	G G G .    
480	C C C .    
481	A A A .    
482	C C C .    
483	T T T .    
484	C C C .    
485	A A A .    
486	T T T .    
487	C C C .    
488	A A A .    
489	A A A .    
490	A A A .    
491	C C C .    
492	A A A .    
493	T T T .    
494	T T T .    
495	A A A .    
496	G G G .    
497	C C C .    
498	A A A .    
499	C C C .    
500	C C C .    
501	A A A .    
502	A A A .    
503	T T T .    
504	C C C .    
505	T T T .    
506	G G G .    
507	T T T .    
508	C C C .    
509	C C C .    
510	G G G .    
511	T T T .    
512	G G G .    
513	T T T .    
514	G G G .    
515	T T T .    
516	A A A .    
517	T T T .    
518	A A A .    
519	A A A .    
520	G G G .    
521	A A A .    
522	A A A .    
523	C C C .    
524	T T T .    
525	G G G .    
526	C C C .    
527	A A A .    
528	T T T .    
529	T T T .    
530	A A A .    
531	C C C .    
532	G G G .    
533	T T T .    
534	T T T .    
535	T T T .    
536	G G G .    
537	T T T .    
538	A A A .    
539	T T T .    
540	A A A .    
541	A A A .    
542	T T T .    
543	T T T .    
544	T T T .    
545	A A A .    
546	G G G .    
547	G G G .    
548	C C C .    
549	C C C .    
550	A A A .    
551	A A A .    
552	G G G .    
553	G G G .    
554	A A A .    
555	A A A .    
556	G G G .    
557	A A A A    
558	A A A A    
559	A A A A    
560	C C C C    
561	A A A A    
562	T T T T    
563	C C C C    
564	T T T T    
565	G G G G    
566	T T T T    
567	T T T T    
568	A A A A    
569	G G G G    
570	T T T T    
571	G G G G    
572	T T T T    
573	A A A A    
574	C C C C    
575	C C C C    
576	A A A A    
577	T T T T    
578	A A A A    
579	T T T T    
580	A A A A    
581	C C C C    
582	C C C C    
583	A A A A    
584	G G G G    
585	C C C C    
586	T T T T    
587	A A A A    
588	A A A A    
589	A A A A    
590	C C C C    
591	T T T T    
592	T T T T    
593	T T T T    
594	T T T T    
595	A A A A    
596	A A A A    
597	A A A A    
598	T T T T    
599	T T T T    
600	A A A A    
601	T T T T    
602	T T T T    
603	T T T T    
604	C C C C    
605	T T T T    
606	A A A A    
607	T T T T    
608	T T T T    
609	A A A A    
610	A A A A    
611	A A A A    
612	G G G G    
613	A A A A    
614	A A A A    
615	T T T T    
616	T T T T    
617	G G G G    
618	G G G G    
619	A A A A    
620	T T T T    
621	T T T T    
622	T T T T    
623	G G G G    
624	T T T T    
625	C C C C    
626	C C C C    
627	T T T T    
628	T T T T    
629	. G G G    
630	. A A A    
631	. T T T    
632	. G G G    
633	. C C C    
634	. C C C    
635	. T T T    
636	. C C C    
637	. T T T    
638	. G G G    
639	. G G G    
640	. A A A    
641	. A A A    
642	. A A A    
643	. T T T    
644	. C C C    
645	. T T T    
646	. C C C    
647	. A A A    
648	. A A A    
649	. T T T    
650	. T T T    
651	. T T T    
652	. G G G    
653	. T T T    
654	. T T T    
655	. T T T    
656	. T T T    
657	. A A A    
658	. T T T    
659	. G G G    
660	. G G G    
661	. C C C    
662	. T T T    
663	. C C C    
664	. A A A    
665	. C C C    
666	. T T T    
667	. T T T    
668	. G G G    
669	. G G G    
670	. A A A    
671	. A A A    
672	. A A A    
673	. T T T    
674	. G G G    
675	. C C -  1	 indel  
676	. A A -  1	 indel  
677	. G G -  1	 indel  
678	. T T -  1	 indel  
679	. T T -  1	 indel  
680	. G G -  1	 indel  
681	. T T T    
682	. T T T    
683	. C C C    
684	. A A A    
685	. A A A    
686	. A A A    
687	. C C C    
688	. A A A    
689	. C C C    
690	. A A A    
691	. G G G    
692	. A A A    
693	. C C C    
694	. T T T    
695	. A A A    
696	. G G G    
697	. C C C    
698	. T T T    
699	. T T T    
700	. A A A    
701	. C C C    
702	. T T T    
703	. C C C    
704	. A A A    
705	. G G G    
706	. T T T    
707	. T T T    
708	. G G G    
709	. C C C    
710	. A A A    
711	. C C C    
712	. T T T    
713	. T T T    
714	. G G G    
715	. G G G    
716	. A A A    
717	. A A A    
718	. A A A    
719	. G G G    
720	. C C C    
721	. A A A    
722	. T T T    
723	. T T T    
724	. G G G    
725	. A A A    
726	. A A A    
727	. T T T    
728	. T T T    
729	. T T T    
730	. A A A    
731	. A A A    
732	. C C C    
733	. C C C    
734	. T T T    
735	. G G G    
736	. T T T    
737	. T T T    
738	. T T T    
739	. T T T    
740	. G G G    
741	. T T T    
742	. A A A    
743	. T T T    
744	. G G G    
745	. G G G    
746	. A A A    
747	. A A A    
748	. T T T    
749	. G G G    
750	. C C C    
751	. T T T    
752	. C C C    
753	. A A A    
754	. C C C    
755	. G G G    
756	. T T T    
757	. A A A    
758	. C C C    
759	. A A A    
760	. A A A    
761	. T T T    
762	. T T T    
763	. G G G    
764	. G G G    
765	. C C C    
766	. A A A    
767	. A A A    
768	. G G G    
769	. T T T    
770	. T T T    
771	. T T T    
772	. C C C    
773	. T T T    
774	. A A A    
775	. T T T    
776	. T T T    
777	. A A A    
778	. A A A    
779	. C C C    
780	. A A A    
781	. T T T    
782	. A A A    
783	. A A A    
784	. T T T    
785	. T T T    
786	. G G G    
787	. A A A    
788	. G G G    
789	. C C C    
790	. A A A    
791	. A A A    
792	. A A A    
793	. T T T    
794	. G G G    
795	. T T T    
796	. C C C    
797	. T T T    
798	. T T T    
799	. C C C    
800	. A A A    
801	. G G G    
802	. A A A    
803	. C C C    
804	. A A A    
805	. C C C    
806	. T T T    
807	. T T T    
808	. T T T    
809	. . A A    
810	. . G G    
811	. . T T    
812	. . A A    
813	. . G G    
814	. . G G    
815	. . A A    
816	. . T T    
817	. . G G    
818	. . G G    
819	. . A A    
820	. . G G    
821	. . A A    
822	. . T T    
823	. . C C    
824	. . T T    
825	. . G G    
826	. . T T    
827	. . C C    
828	. . A A    
829	. . G G    
830	. . T T    
831	. . C C    
832	. . A A    
833	. . G G    
834	. . C C    
835	. . A A    
836	. . T T    
837	. . T T    
838	. . A A    
839	. . T T    
840	. . A A    
841	. . T T    
842	. . A A    
843	. . A A    
844	. . C C    
845	. . C C    
846	. . A A    
847	. . A A    
848	. . T T    
849	. . G G    
850	. . A A    
851	. . C C    
852	. . T T    
853	. . G G    
854	. . T T    
855	. . T T    
856	. . C C    
857	. . A A    
858	. . T T    
859	. . A A    
860	. . C C    
861	. . T T    
862	. . G G    
863	. . A A    
864	. . C C    
865	. . A A    
866	. . G G    
867	. . A A    
868	. . C C    
869	. . A A    
870	. . A A    
871	. . T T    
872	. . T T    
873	. . C C    
874	. . A A    
875	. . T T    
876	. . T T    
877	. . A A    
878	. . A A    
879	. . T T    
880	. . C C    
881	. . T T    
882	. . T T    
883	. . C C    
884	. . A A    
885	. . A A    
886	. . T T    
887	. . A A    
888	. . G G    
889	. . G G    
890	. . G G    
891	. . A A    
892	. . G G    
893	. . C C    
894	. . T T    
895	. . C C    
896	. . A A    
897	. . G G    
898	. . C C    
899	. . T T    
900	. . A A    
901	. . A A    
902	. . C C    
903	. . A A    
904	. . G G    
905	. . A A    
906	. . C C    
907	. . T T    
908	. . A A    
909	. . T T    
910	. . T T    
911	. . T T    
912	. . T T    
913	. . C C    
914	. . T T    
915	. . A A    
916	. . T T    
917	. . C C    
918	. . T T    
919	. . C C    
920	. . T T    
921	. . G G    
922	. . G G    
923	. . T T    
924	. . G G    
925	. . T T    
926	. . T T    
927	. . G G    
928	. . G G    
929	. . T T    
930	. . T T    
931	. . A A    
932	. . T T    
933	. . G G    
934	. . A A    
935	. . A A    
936	. . T T    
937	. . G G    
938	. . C C    
939	. . T T    
940	. . T T    
941	. . A A    
942	. . T T    
943	. . T T    
944	. . T T    
945	. . C C    
946	. . T T    
947	. . A A    
948	. . A A    
949	. . C C    
950	. . C C    
951	. . A A    
952	. . C C    
953	. . T T    
954	. . A A    
955	. . T T    
956	. . T T    
957	. . T T    
958	. . C C    
959	. . A A    
960	. . A A    
961	. . A A    
962	. . T T    
963	. . T T    
964	. . G G    
965	. . A A    
966	. . C C    
967	. . G G    
968	. . T T    
969	. . T T    
970	. . C C    
971	. . A A    
972	. . C C    
973	. . T T    
974	. . G G    
975	. . T T    
976	. . T T    
977	. . A A    
978	. . A A    
979	. . T T    
980	. . T T    
981	. . C C    
982	. . T T    
983	. . C C    
984	. . C C    
985	. . C C    
986	. . A A    
987	. . T T    
988	. . T T    
989	. . A A    
990	. . T T    
991	. . C C    
992	. . C C    
993	. . A A    
994	. . G G    
995	. . G C  1	 transversion  
996	. . . C    
997	. . . C    
998	. . . C    
999	. . . A    
1000	. . . C    
1001	. . . T    
1002	. . . T    
1003	. . . G    
1004	. . . C    
1005	. . . T    
1006	. . . G    
1007	. . . T    
1008	. . . T    
1009	. . . G    
1010	. . . G    
1011	. . . G    
1012	. . . G    
1013	. . . A    
1014	. . . G    
1015	. . . C    
1016	. . . T    
1017	. . . C    
1018	. . . C    
1019	. . . A    
1020	. . . C    
1021	. . . T    
1022	. . . G    
1023	. . . A    
1024	. . . T    
1025	. . . C    
1026	. . . A    
1027	. . . C    
1028	. . . C    
1029	. . . C    
1030	. . . A    
1031	. . . G    
1032	. . . T    
1033	. . . T    
1034	. . . T    
1035	. . . A    
1036	. . . C    
1037	. . . A    
1038	. . . A    
1039	. . . T    
1040	. . . G    
1041	. . . A    
1042	. . . A    
1043	. . . T    
1044	. . . A    
1045	. . . A    
1046	. . . T    
1047	. . . G    
1048	. . . C    
1049	. . . A    
1050	. . . A    
1051	. . . T    
1052	. . . T    
1053	. . . G    
1054	. . . C    
1055	. . . C    
1056	. . . G    
1057	. . . T    
1058	. . . G    
1059	. . . T    
1060	. . . G    
1061	. . . A    
1062	. . . C    
1063	. . . C    
1064	. . . T    
1065	. . . C    
1066	. . . A    
1067	. . . T    
1068	. . . A    
1069	. . . T    
1070	. . . G    
1071	. . . T    
1072	. . . T    
1073	. . . G    
1074	. . . C    
1075	. . . C    
1076	. . . C    
1077	. . . C    
1078	. . . C    
1079	. . . A    
1080	. . . T    
1081	. . . T    
1082	. . . C    
1083	. . . T    
1084	. . . T    
1085	. . . G    
1086	. . . A    
1087	. . . G    
1088	. . . C    
1089	. . . T    
1090	. . . A    
1091	. . . C    
1092	. . . G    
1093	. . . T    
1094	. . . G    
1095	. . . A    
1096	. . . T    
1097	. . . G    
1098	. . . A    
1099	. . . C    
1100	. . . C    
1101	. . . G    
1102	. . . G    
1103	. . . G    
1104	. . . G    
1105	. . . G    
1106	. . . T    
1107	. . . G    
1108	. . . G    
1109	. . . G    
1110	. . . C    
1111	. . . G    
1112	. . . G    
1113	. . . C    
1114	. . . T    
1115	. . . C    
1116	. . . C
```
